# Molecular systematics and the evolution of mycoheterotrophy of tribe Neottieae (Orchidaceae, Epidendroideae)

**DOI:** 10.3897/phytokeys.94.21346

**Published:** 2018-01-29

**Authors:** Ting Zhou, Xiao-Hua Jin

**Affiliations:** 1 State Key Laboratory of Systematic and Evolutionary Botany, Institute of Botany, Chinese Academy of Sciences, Beijing 100093, P. R. China

**Keywords:** Generic delimitation, Molecular phylogenetics, Mycoheterotrophy, Neottia, peloric form

## Abstract

Neottieae comprise about 150–200 species and are distributed mainly in temperate and subtropical zones of the northern hemisphere. Mycoheterotrophy is common in Neottieae. Based on three DNA markers and a broad sampling of Neottieae, these results indicate that Neottieae is strongly supported as monophyletic and *Palmorchis* is sister to the remaining genera of Neottieae. *Holopogon* and *Neottia* s.s. are deeply nested within *Listera*. The habit of leafless mycotrophy has independently evolved at least three times in Neottieae, one in *Cephalanthera*, another in *Neottia* s.l. and the third in the clade formed by *Limodorum* and *Aphyllorchis*.

## Introduction

Neottieae Lindl. is a small tribe in Orchidaceae, comprising about 150–200 species and distributed mainly in temperate and subtropical zones of the northern hemisphere with a few species extending into tropical alpine regions (Chen et al. 2009; Dressler 1981; Jin 2014; Jin and Pang 2016; Pridgeon et al. 2005). Most phylogenetic reconstructions indicate that Neottieae is monophyletic and one of the basal groups of the subfamily Epidendroideae (Chase et al. 2015; Feng et al. 2016; Freudenstein and Chase 2015; Freudenstein et al. 2004; Gorniak et al. 2010; van den Berg et al. 2005; Xiang et al. 2012). Recent literature indicates that the habits of mixotrophy and of mycoheterotrophy are common in Neottieae (Gebauer and Meyer 2003; Girlanda et al. 2006; Jacquemyn et al. 2015; Julou et al. 2005; Liebel et al. 2010; Tesitelova et al. 2015; Yagame et al. 2016). Pridgeon et al. (2005) stated that one of the remarkable evolutionary trends in Neottieae is the repeated transition from photosynthetic autotrophy to obligate mycoheterotrophy. Some mixotrophic orchids, such as *Cephalanthera damasonium*, *Epipactis* spp. and *Limodorum abortivum*, obtain carbon from their mycorrhizal fungi and through photosynthesis (Girlanda et al. 2006; Julou et al. 2005; Liebel et al. 2010), while some species, such as *Aphyllorchis caudata* and *Cephalanthera exigua*, are fully mycoheterotrophic. Julou et al. (2005) proposed the evolution of mycoheterotrophy in Neottieae in three successive transitions: first a ‘mycorrhizal shift’ to ectomycorrhizas fungi allowing mixotrophic nutrition; second a transition to high specificity; and third a transition to fully heterotrophic nutrition.

The aims of the present study are 1) to analyse phylogenetic interrelationships within Neottieae using evidence from molecular data (chloroplast *mat*K, *rbcL* and nuclear ITS) and 2) to understand the evolutionary pattern of the mycoheterotrophic habit in Neottieae.

## Material and methods

### Taxon sampling

Sixty-eight species were included in this study, representing eight genera of Neottieae: *Aphyllorchis*, *Cephalanthera*, *Epipactis*, *Holopogon*, *Limodorum*, *Listera*, *Neottia* and *Palmorchis*. Outgroups include three species from tribe Orchideae: *Ophrys insectifera*, *Ophrys apifera* and *Serapias cordigera*. Chloroplast DNA (specifically *rbcL* and *matK*) and nuclear ITS were analysed. Voucher information and GenBank accession numbers are shown in Supplementary material 1. New sequences are in Supplementary material 4–6.

### DNA extraction, amplification and sequencing

Total genomic DNA was isolated from silica-gel-dried materials using a Plant Genomic DNA Kit (Beijing Biomed Co., LTD, Beijing, China). For this study, three markers (the coding plastid gene *matK*, *rbcL*) and the nuclear ribosomal DNA internal transcribed spacers (ITS) were used. The PCR and sequencing primers for *matK*, *rbcL* and ITS are listed in Supplementary material 2. The selected DNA regions were amplified by using a standard polymerase chain reaction (PCR). The sequencing reactions were performed by using the ABI Prism Bigdye Terminator Cycle Sequencing Kit (Applied Biosystems, ABI).

### Phylogenetic analysis

Sequences were aligned using the default parameters in ClustalX v1.83 (Thompson et al. 1997) and manually adjusted with BioEdit v5.0.9 (Hall 1999). Alignments are listed in Supplementary material 4–6. Phylogenetic analyses of the combined dataset were carried out using parsimony (PAUP* v4.0b10) (Swofford 2003) and Bayesian Inference (BI; MrBayes v3.2.0) (Ronquist and Huelsenbeck 2003). Parsimony heuristic searches were performed with 1000 random sequence addition replicates, tree-bisection-reconnection (TBR) branch swapping, MulTrees in effect and steepest descent off, saving all minimum length trees (MULPARS on). Internal branch support under maximum parsimony (MP) was estimated by using 1000 bootstrap (BS) replicates; the starting trees were obtained by random addition with ten replicates for each replication, TBR branch swapping and MULPARS in effect.

For BI analyses, the data were partitioned a priori on the basis of gene identity and, for coding regions, codon position. Based on Bayes factors, the partitioning strategy (*rbcL*, *matK*) was identified as optimal for these data and was applied in all subsequent Bayesian analyses. Initial analyses providing data for comparison of the different partition strategies were run for 3000000 generations and analyses applying the final best-fit model were run for 5000000 generations. Runs were started from a random tree sampled every 1000 generations of the MCMC chain, with default priors and the option prset/ratepr set as variable. Each parameter estimation, obtained from the results of two runs, was checked in Tracer v1.5 (http://tree.bio.ed.ac.uk/software/tracer) to ascertain whether they had obtained a proper effective sample size and to verify that the stationary state had been reached. Trees from the ﬁrst 10% of generations were discarded as burn-in. The remaining trees were combined to build a 50% majority-rule consensus tree. Bayesian Inference was run on CIPRES (Miller et al. 2010).

## Results

### Sequences characteristics

In this study, 159 new sequences were obtained (60 ITS, 48 *matK*, 51 *rbcL*). In the overall matrix, the combined dataset of three markers comprised 3817 aligned nucleotides: 730 bp from ITS and 3087 bp from chloroplast regions; 24% of the combined alignment sites were parsimony-informative. The alignments of each matrix and their properties are summarised in Supplementary material 3–6.

### Phylogenetic analyses

As the partition homogeneity test for plastid DNA + ITS shows there were no strongly supported incongruent results in the datasets (*P* = 0.17), the datasets for simultaneous analyses were therefore combined.

**Figure 1.**
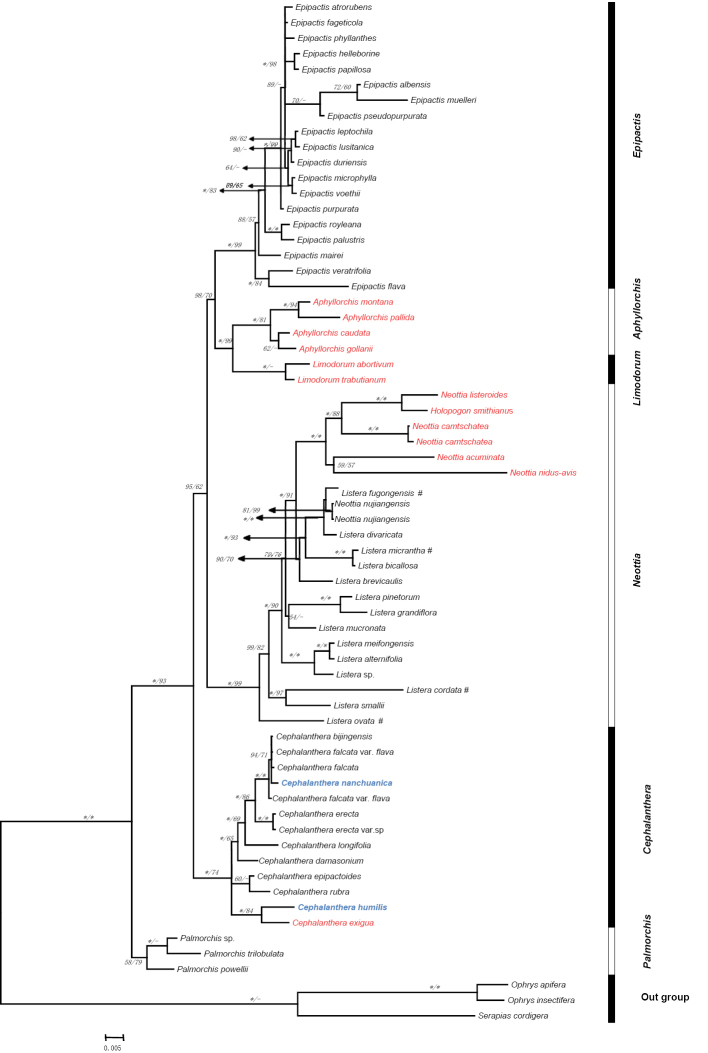
Phylogram obtained from Bayesian Inference analysis of combined nrDNA ITS, matK and rbcL data. Numbers at nodes are Bayesian posterior probabilities and bootstrap percentages (≥ 50%), respectively. ‘‘–’’ indicates that the node was not supported in MP analysis, asterisk (*) represent 100% support and red colour denotes species that are mycotrophic herbs.

Phylogenetic relationships based on the ITS data had a better resolution than the two combined plastid DNA data (results not shown here). Based on the combined ITS and plastid DNA data, these findings are consistent in the overall topology of the trees produced with maximum parsimony (MP) and Bayesian Inference (BI) methods, except for a few of the collapsed nodes. Bootstrap values (BS) were often lower than the Posterior Probability (PP) from the Bayesian analysis. The BI topology from the combined dataset was chosen as the primary tree for discussion of phylogenetic relationships (Figure 1; the MP strict consensus tree is not shown).

The results indicated that the tribe Neottieae can be subdivided into five clades:

Clade I consists of sampled species of *Epipactis* and all the species can be subdivided into 2 subclades: subclade I includes 17 species (PP = 88, BP = 57); subclade II includes *Epipactis veratrifolia* and *Epipactis flava* (PP = 100, BP = 83).

Clade II consists of sampled species of *Aphyllorchis* and *Limodorum* with strong support (PP = 100, BP = 99). *Aphyllorchis* is moderately supported as monophyletic (PP = 100, BP = 81) and is subdivided into two groups, a temperate group and a tropical group.

Clade III includes sampled species of *Holopogon*, *Listera*, *Neottia* with strong support (PP = 100, BP = 99). All sampled mycoheterotrophic species of *Holopogon* and *Neottia* form a monophyletic subclade nested within *Listera* with strong support (PP = 100, BP = 100) and sister to an autotrophic and alpine group. *Neottia alternifolia* is sister to *N. morrisonicola* with strong support (PP = 100, BP = 99).

Clade IV includes sampled species of *Cephalanthera* with moderate support (PP = 100, BP = 75) and forms a polytomy with three groups: an Eastern Asian autotrophic group with 4–5 species (pp = 1.00, BP=65), a holomycotrophic group with 2 species (pp = 1.00, BP = 84) and a Central Asian Group with 2 species.

Clade V includes sampled species of *Palmorchis* (PP = 100, BP = 100) and is sister to the other four clades.

## Discussion

### Phylogenetic interrelationships within Neottieae

There are some discussions about the phylogenetic relationships in Neottieae and its alliance (Feng et al. 2016; Pridgeon et al. 2005; Xiang et al. 2012), most of these interrelations being supported by the authors’ results. However, many new relationships within Neottieae were discovered.

***Epipactis***: Although *Epipactis* is a small to middle size genus with 15–75 species, there is much debate about species delimitation in this genus (Squirrell et al. 2002). Although these results indicate that *Epipactis* is strongly supported as monophyletic and is sister to clade II formed by *Aphyllorchis* and *Limodorum*, the infrageneric system needs to be re-evaluated. Two species of sect. Arthrochilium, *Epipactis veratrifolia* and *E. flava*, form a well resolved clade that is sister to the remaining Epipactis species. Sect. Epipactis is deeply nested within sect. Arthrochilum and is supported as a monophyletic clade (PP = 1.00, BS = 95). Despite its morphological homogeneity, sect. Arthrochilum is paraphyletic as sect. Epipactis is deeply embedded within it.

***Aphyllorchis* and *Limodorum***: The sister relationship between *Aphyllorchis* and *Limodorum* is supported by the shared holomycotrophic habit and several morphological characters, such as the long and slender column and two powdery pollinia with viscidium (Pridgeon et al. 2005). However, these two genera can be distinguished easily by the lip morphology and by their distribution pattern. *Limodorum* has an entire labellum with a spur and is restricted to central and southern Europe, southwest Asia and north Africa, whereas *Aphyllorchis* has a more or less lobed labellum without spur and is restricted to the area ranging from the eastern part of Asia to Australia. The four sampled species of *Aphyllorchis* are subdivided into two groups; one is of two species (*A. pallida*, *A. montana*) restricted to montane forest in tropical Asia, while the other is distributed mainly in subtropical regions.

***Neottia* s.l.**: *Neottia* s.l. is monophyletic with strong support (PP = 1.00, BS = 99). Several widespread temperate species, *Neottia ovata*, *N. cordata* + *Neottia smallii*, are resolved as the successive basal groups in *Neottia*, while the mycotrophic and alpine taxa are nested deeply within *Neottia* (Figure 1). Four sampled mycotrophic taxa form a clade with strong support (PP = 1.00, BP = 88), sister to the alpine group (including *Neottia brevicaulis*). The relationship between *Neottia alternifolia*, *N. morrisonicola* and *Neottia* sp. (Jin 11279) is supported by morphological characters, such as two more or less alternate leaves in the middle of the stem, apex of lip shallowly notched or emarginate and column short. *Neottia bicallosa* is sister to *N. micrantha* (PP = 1.00, BS = 100) supported by morphological characters, such as two prostrate leaves, three-lobed lip and mid-lobe dentate, column short. *Neottia camtschatea* is sister to the group formed by *N. listeroides + Holopogon* with strong support (PP = 1.00, BS = 88).

***Cephalanthera***: *Cephalanthera* is moderately supported as monophyletic and resolved as sister to Clade I + Clade II + Clade III with weak support. Peloric forms are common in *Cephalanthera*, such as *C. humilis*, *C. nanchuanica*, C. falcate var. flava and C. erecta var. lanceolata. The results indicated that such peloric forms have independently evolved at least three times in *Cephalanthera* (Figure 1).

***Palmorchis***: There is some debate about the phylogenetic treatment of *Palmorchis*. Dressler (1993) treated *Palmorchis* as a distinct tribe, Palmorchideae, while Chase et al. (2003) placed it in Neottieae and was followed by later authors. Recent results of molecular evidence indicated that *Palmorchis* is sister to the remaining genera of Neottieae (Cameron et al. 1999; Freudenstein et al. 2004; Xiang et al. 2012) and the authors’ results agree well with these results. Morphologically, *Palmorchis* is isolated in Neottieae by having reed-like stems, petiolate and plicate leaves, column more or less adnate to lip, four granular or subceraceous pollinia without caudicles or stipes (Dressler 1993). *Palmorchis* is restricted to Central and South America, where no other members of Neottieae s.s. occurs.

### Evolution of mixotrophic and mycoheterotrophic habit

Leafless mixotrophic/mycoheterotrophic orchids of Neottieae can be subdivided into two kinds. One is obligate mycoheterotrophic, such as *Cephalanthera exigua* and *Neottia nidus-avis*, characterised by achlorophyllous plants, fleshy roots without root hairs. The other is mixotrophic, such as *Limodorum abortivum*, characterised by leafless but more or less green plants, roots elongate, more or less hairy. The results indicate that the habit of leafless mixotrophic/mycoheterotrophic orchids has independently evolved at least three times in Neottieae, in Clade II (*Cephalanthera*), Clade III (*Neottia* s.l.) and Clade V (*Limodorum + Aphyllorchis*) respectively.

Julou et al. (2005) suggested the evolution of a three-step transition to mycoheterotrophy via mixotrophy. The results indicated an interesting evolution pattern of mixotrophic/mycoheterotrophic orchids, which may have evolved independently within each clade. Two mycoheterotrophic species (*C. humilis*, *C. exigua*) and two mixotrophic ones (*C. damasonium*, *C. longifolia*) are nested within *Cephalanthera*. In Clade V, the mixotrophic genus *Limodorum* is sister to the mycoheterotrophic genus *Aphyllorchis*. Recent results indicate that mixotrophy has been confirmed in *Platanthera minor* (Yagame et al. 2012) and that specificity of mycoheterotrophic orchis-fungus association is low (Roy et al. 2009). It seems that mycoheterotrophy and mixotrophy could evolve from autotrophic ancestors independently. However, this hypothesis needs to be further tested.

## Supplementary materials

Supplementary material 1 Table 1 Link: https://doi.org/10.3897/phytokeys.94.21346.suppl1 Data type: Word document. Explanation note: List of taxa, vouchers and GenBank accession numbers downloaded from NCBI. Newly sampled sequences are in Supplementary material 4–6. This is an open access article distributed under the terms of the Creative Commons Attribution License (CC BY 4.0), which permits unrestricted use, distribution, and reproduction in any medium, provided the original author and source are credited.Ting Zhou, Xiao-Hua Jin

Supplementary material 2 Table 2 Link: https://doi.org/10.3897/phytokeys.94.21346.suppl2 Data type: Word document. Explanation note: A list of primers used for PCR and sequences in this study. This is an open access article distributed under the terms of the Creative Commons Attribution License (CC BY 4.0), which permits unrestricted use, distribution, and reproduction in any medium, provided the original author and source are credited.Ting Zhou, Xiao-Hua Jin

Supplementary material 3 Table 3 Link: https://doi.org/10.3897/phytokeys.94.21346.suppl3 Data type: Word document. Explanation note: Analyses of datasets. This is an open access article distributed under the terms of the Creative Commons Attribution License (CC BY 4.0), which permits unrestricted use, distribution, and reproduction in any medium, provided the original author and source are credited.Ting Zhou, Xiao-Hua Jin

Supplementary material 4 Alignment of ITS Link: https://doi.org/10.3897/phytokeys.94.21346.suppl4 Data type: Fasta file. Explanation note: Alignment of ITS. This is an open access article distributed under the terms of the Creative Commons Attribution License (CC BY 4.0), which permits unrestricted use, distribution, and reproduction in any medium, provided the original author and source are credited.Ting Zhou, Xiao-Hua Jin

Supplementary material 5 Alignment of matK Link: https://doi.org/10.3897/phytokeys.94.21346.suppl5 Data type: Fasta file. Explanation note: Alignment of matK. This is an open access article distributed under the terms of the Creative Commons Attribution License (CC BY 4.0), which permits unrestricted use, distribution, and reproduction in any medium, provided the original author and source are credited.Ting Zhou, Xiao-Hua Jin

Supplementary material 6 Alignment of rbcL Link: https://doi.org/10.3897/phytokeys.94.21346.suppl6 Data type: Fasta file. Explanation note: Alignment of rbcL. This is an open access article distributed under the terms of the Creative Commons Attribution License (CC BY 4.0), which permits unrestricted use, distribution, and reproduction in any medium, provided the original author and source are credited.Ting Zhou, Xiao-Hua Jin
